# Gene buddies: linked balanced polymorphisms reinforce each other even in the absence of epistasis

**DOI:** 10.7717/peerj.5110

**Published:** 2018-06-28

**Authors:** Jacob A. Tennessen

**Affiliations:** Department of Integrative Biology, Oregon State University, Corvallis, OR, USA

**Keywords:** Balancing selection, Epistasis, Simulation, Population genetics, Linkage

## Abstract

The fates of genetic polymorphisms maintained by balancing selection depend on evolutionary dynamics at linked sites. While coevolution across linked, epigenetically-interacting loci has been extensively explored, such supergenes may be relatively rare. However, genes harboring adaptive variation can occur in close physical proximity while generating independent effects on fitness. Here, I present a model in which two linked loci without epistasis are both under balancing selection for unrelated reasons. Using forward-time simulations, I show that recombination rate strongly influences the retention of adaptive polymorphism, especially for intermediate selection coefficients. A locus is more likely to retain adaptive variation if it is closely linked to another locus under balancing selection, even if the two loci have no interaction. Thus, two linked polymorphisms can both be retained indefinitely even when they would both be lost to drift if unlinked. While these results may be intuitive, they have important implications for genetic architecture: clusters of mutually reinforcing genes may underlie phenotypic variation in natural populations, and such genes cannot be assumed to be functionally associated. Future studies that measure selection coefficients and recombination rates among closely linked genes will be fruitful for characterizing the extent of this phenomenon.

## Introduction

Balancing selection is an evolutionary process that maintains more than one allele at a locus for longer than would be expected under genetic drift alone (Fijarczyk & Babik, 2015). Numerous examples of balanced polymorphisms are well known, related to infectious disease (Pasvol, Weatherall & Wilson, 1978; Hughes & Nei, 1988; Tennessen & Blouin, 2008), reproduction (Wright, 1939; Käfer, Marais & Pannell, 2017), and other traits (reviewed in Llaurens, Whibley & Joron, 2017). Balancing selection can operate via several distinct mechanisms, including overdominance or heterozygote advantage, frequency-dependent selection, or spatiotemporally varying selection (Tennessen & Blouin, 2008). While early population geneticists proposed widespread balancing selection to explain polymorphisms (Lewontin & Hubby, 1966), this view fell out of favor with the rise of the neutral theory (Kimura, 1983). However, without validating the pan-adaptationism of earlier decades, the importance of balancing selection is being increasingly recognized (Sellis et al., 2011; Delph & Kelly, 2014). In particular, the deluge of population genomic data from humans, accompanied by new sophisticated methods for detecting balancing selection, has recently revealed numerous candidate polymorphisms maintained by selection in our species (Ségurél et al., 2012; Leffler et al., 2013; Key et al., 2014; Teixeira et al., 2015; Bitarello et al., 2018). In taxa such as Drosophila with relatively large population sizes, adaptively maintained polymorphisms may be even more prevalent (Bergland et al., 2014; Croze et al., 2017).

For an adaptive polymorphism to survive, balancing selection must overcome genetic drift (Sutton et al., 2011), which is primarily determined by the effective population size (*N_e_*). When *s*, the selective advantage of a genotype, is much less than 1/*N_e_*, the polymorphism is effectively neutral and it is eventually lost, whereas if *s* is much greater than 1/*N_e_*, the effects of selection will govern evolution (Ohta, 1992). Thus, when *s* is close in magnitude to 1/*N_e_*, the opposing forces of balancing selection and drift nearly cancel out, and the fate of a balanced polymorphism may be more heavily influenced by other factors, including selection acting on linked sites. Linkage disequilibrium (LD) between physically adjacent loci has been recognized as an important factor determining evolutionary outcomes, and thus a haplotype block of several loci in LD, rather than an individual locus, may be thought of as the unit of selection (Casillas & Barbadilla, 2017). The extent of LD between two linked loci depends on the recombination rate (*r*), *N_e_*, and other evolutionary forces. For balancing selection, this haplotype framework raises the question of if, and how, genetic variation at one locus affects the probability that adaptive variation will be maintained at linked loci. It is well known that balancing selection increases the coalescence time at linked neutral sites (Charlesworth, Nordborg & Charlesworth, 1997; Nordborg, 1997; Charlesworth, 2006), but linkage to non-neutral sites, which themselves may be under balancing selection, has been less explored.

One obvious mechanism is epistasis: if only certain combinations of alleles at two linked loci yield high fitness, then allele frequencies at one locus will depend on those at the other locus, resulting in epistatically interacting “supergenes” (reviewed in Thompson & Jiggins, 2014; Llaurens, Whibley & Joron, 2017). Intuitively, if the fitness effect of one locus depends on the genotype at a second, linked locus, then not only might selection favor LD between these loci, but the strength of selection on the resulting multi-locus haplotype may be greater than it would be for either locus in isolation. In deterministic models, linkage only affects equilibrium allele frequencies when there is epistasis in an additive sense (Lewontin & Kojima, 1960; Karlin & Feldman, 1970; Nagylaki, 2009). While selection can still maintain polymorphism in the absence of epistasis, a locus receives no selective benefit from being linked to other balanced polymorphisms, at least with respect to deterministic equilibrium allele frequencies. Thus, explanations for clusters of balanced polymorphisms such as sex chromosomes (Bergero & Charlesworth, 2009), the major histocompatibility complex (Slatkin, 2000; Trowsdale, 2002; Makino & McLysaght, 2008), or generalized quantitative trait architecture (Karlin & Feldman, 1970; Draghi & Whitlock, 2015) often implicitly assume epistasis. Epistasis is ubiquitous in genotype/phenotype relationships, and evidence of epistasis between closely-linked loci has been observed in some cases (Rice, 1987; Gregersen et al., 2006). However, in many systems there is little empirical evidence that co-adaptation among linked genes maintains polymorphism (Ohta, 1982; Navarro & Barton, 2002; Wegner, 2008; Corbett-Detig & Hartl, 2012). In particular, while sex chromosomes are the most striking examples of supergenes both in their taxonomic ubiquity and their extreme heteromorphism, the degree to which epistasis (i.e., sexual antagonism) shapes sex chromosome evolution remains contentious (Fry, 2009; Ironside, 2010; Pennell et al., 2015; Branco et al., 2017).

If previous work has overemphasized the importance of epistasis in shaping linked adaptive polymorphisms, it is worth exploring the dynamics of linked polymorphisms with no functional relationship. In finite populations, genetic drift may prevent weakly selected loci from reaching equilibrium conditions, and in these cases, linkage can have a nontrivial effect. Because of this, the extensive results from deterministic models of non-epistatic loci (Lewontin & Kojima, 1960; Karlin & Feldman, 1970; Karlin, 1975) may underestimate the evolutionary importance of linkage. Polymorphisms maintained by balancing selection could become clustered in the genome not because they interact, but because selection acts more efficiently on multi-locus haplotypes than on individual loci. Here, I explore the extent to which polymorphism at a locus under balancing selection depends on other independent instances of balancing selection, acting for unrelated reasons on linked loci.

## Methods

### BalanceLinkage simulations

In order to test whether balanced polymorphisms can show mutual reinforcement in the absence of epistasis, I used forward-time simulations as implemented by a custom Perl script, *BalanceLinkage.pl*. I simulated the evolutionary dynamics of two linked polymorphisms under balancing selection in a finite population without epitasis. The fitness of a diploid individual was 1 for double homozygotes, and larger by *s* for each heterozygous locus, such that double heterozygotes had a fitness of 1 + 2*s*. Thus, the model was consistent with overdominance, which is mathematically equivalent to some forms of frequency-dependent selection (Takahata & Nei, 1990). Following the format of Lewontin & Kojima (1960), this model conforms to the formula

(1) $$a - b - c + d = 0$$

where *a* is the fitness of double homozygotes (here 1), *b* and *c* are the fitnesses of each single heterozygote (here both 1 + *s*), and *d* is the fitness of double heterozygotes (here 1 + 2*s*). Thus, there is no epistasis as defined by Lewontin & Kojima (1960). Because fitness is calculated by summing the selection coefficients, here fitnesses are considered additive. I do not consider the scenario where fitnesses are non-epistatic but only in the multiplicative sense (i.e., if the fitness of double heterozygotes includes an *s*^2^ term). Multiplicative fitnesses are known to maintain LD even in deterministic models (Karlin, 1975; Hastings, 1981), and intuitively, if double heterozygotes have an exponential fitness boost relative to single heterozygotes, one would expect a synergetic effect on the retention of polymorphism. Results from simulations with multiplicative fintesses are similar to those presented here (see below), and in principle should lead to at least as much mutual reinforcement between loci, if not more.

I set the initial frequencies of all four haplotypes to 25%. That is, both polymorphisms occurred at 50% frequency and were not in LD with each other. I set the population size at 1,000, thus *N_e_* = 2*N* = 2,000. I set *s* to various values between 0.002 and 0.01, thus *N_e_s* ranged from 4 to 20 (Table S1). I set *ρ*, the population-scaled recombination rate (= 4*N_e_r*), to 0 (perfect linkage), 4,000 (effectively negligible linkage, as half of all gametes will be recombinant each generation), or various values ranging from 0.005 to 20 (Table S1). Simulations were run forward in time for 100,000 (= 50 *N_e_*) generations, with 1,000 replicates of each parameter combination. I recorded how often one or both polymorphisms were retained. For all combinations of parameters, I recorded the median time to fixation (MTF) for a polymorphism. In order to test for a significant effect of *ρ* on MTF, I ran linear regression of log(MTF) as a function of log(*ρ*). I also recorded the number of simulations (out of 1,000 replicates) for which both polymorphisms were retained for all 50 *N_e_* generations, and I ran linear regression of the log of this value plus one (potential range log(1) to log(1001)) as a function of log(*ρ*).

All simulations can be run using the *BalanceLinkage.pl* Perl script available at https://github.com/jacobtennessen/GeneBuddies. This forward-time simulator allows users to set the selection coefficient (default = 0.005), the number of diploid individuals (default = 1,000), the recombination rate *ρ* (default = 0), the number of generations (default = 100,000), and the number of replicate simulations (default = 1,000). Two polymorphic loci under balancing selection are considered. Though the recombination rate may vary, no intervening sequence is simulated. Mutations are not simulated, as the loci are polymorphic from the start. Each generation, diploid parents are randomly generated based on the existing haplotype frequencies. For heterozygous parents, the relative number of gametes contributed to the following generation is increased based on the selection coefficient. For doubly heterozygous parents, a proportion of gametes (determined by the recombination rate) are replaced with recombined gametes. The output lists how many simulations produced each of five possible results (“Fixed”, i.e., no retained polymorphism; “Similar Two”, i.e., two retained haplotypes that are the same at one locus; “Opposite Two”, i.e., two retained haplotypes that differ at both loci; “Three”, i.e., three retained haplotypes; or “All”, i.e., all four haplotypes retained). If fewer than half of all simulations retained polymorphism at the first locus, the MTF is also reported.

### SLiM simulations

In order to independently confirm the main results, I performed similar simulations using SLiM v. 2.6 (Haller & Messer, 2017). SLiM is a versatile forward-time molecular evolution simulator. It cannot model the aforementioned selection scenario exactly, but it can produce a close approximation. The fitness of a heterozygote *w*_het_ is calculated as

(2) $${w_{{\rm{het}}}} = w(1 + d{s_{{\rm{hom}}}})$$

where *w* is the “background” fitness as determined by any other loci, *d* is the dominance coefficient, and *s*_hom_ is the selection coefficient for homozygotes, such that *ds*_hom_ is equivalent to *s* as defined above. Thus, fitnesses are multiplied rather than added. Furthermore, SLiM cannot model symmetrical overdominance directly, since *s* determines the difference in fitness between the two homozygous genotypes, which therefore cannot be equal if *s* is nonzero. However, the dominance coefficient has no upper bound, so overdominance can be approximated by setting *d* to a very large value above 1, and *s*_hom_ to a very small value such that *ds*_hom_ yields the desired heterozygote-specific selection coefficient, while the difference in fitness between homozygous genotypes is negligible. Thus, I set *d* to 1,000,000 and adjusted *s*_hom_ accordingly. I simulated a 2 bp sequence with various recombination rates between the two sites. As before, I simulated 1,000 replicates of a diploid populations with 1,000 individuals for 100,000 generations. Instead of setting all four haplotype combinations to exactly 25% frequency as before, in the first generation I randomly mutated half of all genomes at the first locus, and independently mutated another random half at the second locus. No subsequent mutations were permitted. As before, I recorded how many simulations retained polymorphism for the duration of the simulation as well as the MTF. Because the *SLiM* analysis was intended as a confirmation of the previous analysis, I did not test all parameter combinations, but evaluated three selection coefficients (*N_e_ds*_hom_ = 5, 10, or 20) and four recombination rates (*ρ* = 0.01, 0.1, 1, or 10).

## Results

### BalanceLinkage simulations

**Figure 1:**
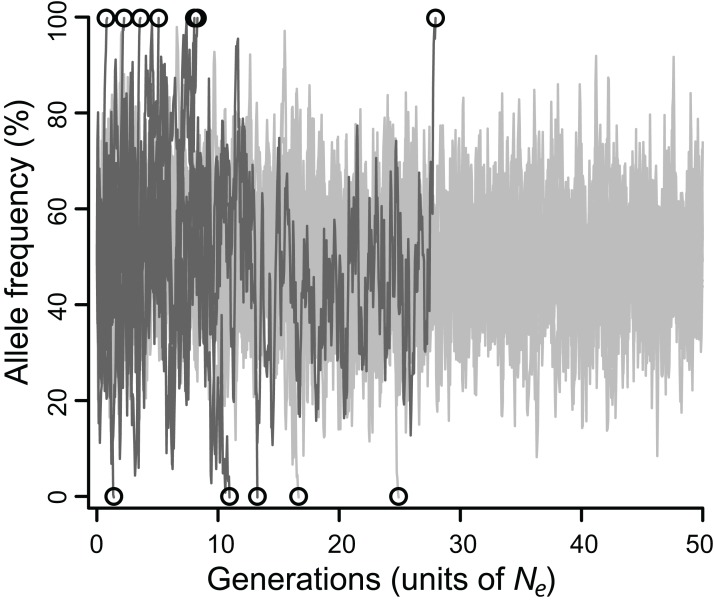
Allele frequencies across 100,000 generations (= 50 *N*_e_) at a locus under balancing selection linked to another locus under balancing selection, without epistasis. Twenty simulations are depicted, all with intermediate selection (*N*_e_*s* = 10), of which 10 have perfect linkage (i.e., no recombination; light grey), and 10 have negligible linkage (dark gray). Circles mark the moment of fixation. With perfect linkage, approximately 80% of simulated loci retain their polymorphism for the duration of the simulation (eight out of 10 do so here), while under negligible linkage, nearly all loci fix well before 50 *N*_e_ generations (all 10 do so here).

The efficacy of selection varied widely depending on model parameters. Allele frequencies typically fluctuated substantially, eventually fixing (Fig. 1), but the fates of balanced polymorphism depended strongly on recombination rates, especially at intermediate selection strengths. Although all simulations started with 50% allele frequencies, the highly variable allele frequencies observed over the course of most simulations (Fig. 1) suggest that similar results would be obtained for any intermediate initial allele frequency (i.e., at least 20%), and probably for rarer initial frequencies as well.

**Figure 2:**
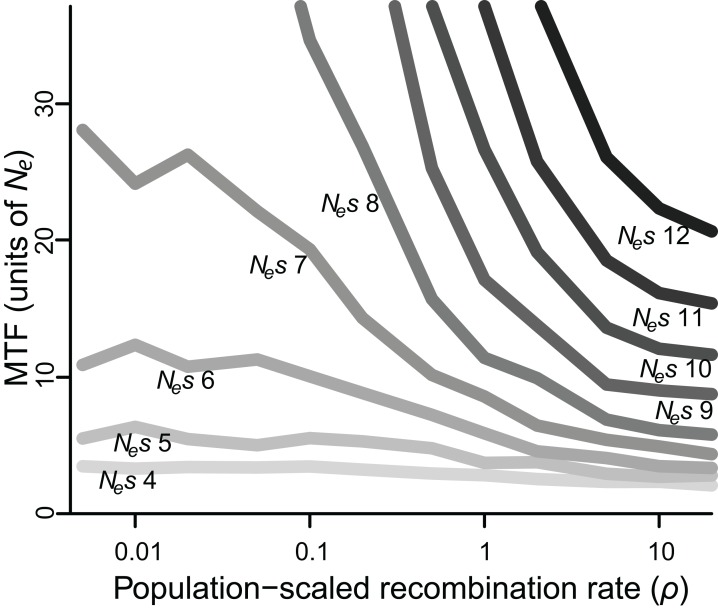
Median time to fixation (MTF) for a polymorphism linked to another polymorphism, for varying values of selection (*N*_e_*s* ranging from four to 12) and recombination (*ρ* ranging from 0.005 to 20). Fixation takes longer when recombination is low, although the magnitude of this effect depends on the strength of selection. Results for *N*_e_*s* values larger than 12 are not shown, as most polymorphisms endured for all 50 *N*_e_ generations across most values of *ρ.*

When selection was weak (*N_e_s* ≤ 5), polymorphism was lost relatively quickly in 2–7 *N_e_* generations regardless of recombination rate, whereas if selection was strong (*N_e_s* ≥ 15), polymorphism was typically retained for the duration of the simulated 50 *N_e_* generations (Table S1). However, for all values of *N_e_s*, lower values of *ρ* were associated with greater retention of polymorphism (Fig. 2). Specifically, for *N_e_s* values of 12 and under, there was a significantly negative relationship between *ρ* and MTF (*P* < 0.05; Table 1). The strongest effect was observed for *N_e_s* of 8, such that reducing *ρ* by 50% resulted in a 29% increase in MTF (*P* < 10^−5^; Table 1), and thus MTF varied by an order of magnitude depending on *ρ*. A similar trend occurred for higher values of *N_e_s*, but since polymorphism was typically retained for all 50 *N_e_* generations even at high *ρ* values, the effect could not be rigorously quantified.

**Table 1:** Values of *N*_e_*s* for which *ρ* had a significant effect on median time to fixation (MTF) over 50 *N_e_* generations.

*N_e_s*	% increase in MTF if ρ is halved	*P*-value
4	4.4	<10^−5^
5	7.3	<10^−5^
6	12.7	<10^−5^
7	19.0	<10^−5^
8	29.4	<10^−5^
9	28.7	<10^−3^
10	25.3	<0.01
11	22.7	<0.01
12	20.1	<0.05

**Figure 3:**
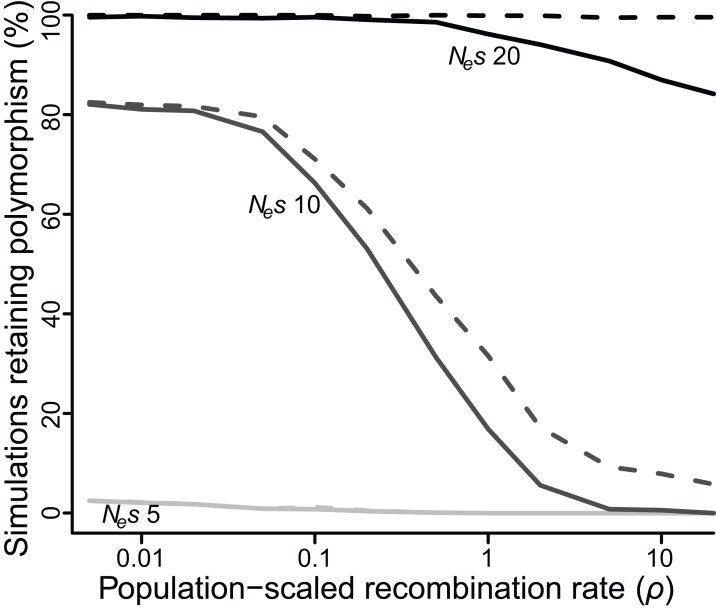
Proportion of simulations where polymorphism is retained after 50 *N*_e_ generations, for varying values of selection (*N*_e_*s* of five, 10 or 20) and recombination (ρ ranging from 0.005 to 20). Solid lines = polymorphism retained at both loci; dashed lines = polymorphism retained at one or both loci.

As an alternative means to infer the effect of *ρ*, I recorded the percentage of simulations that retained polymorphism at one or both loci for at least 50 *N_e_* generations across all values of *ρ* (Fig. 3). At low values of *N_e_s* (≤5), very few polymorphisms (≤2.5%) persisted for 50 *N_e_* generations for any value of *ρ*, but there was still a significant effect of *ρ*. At *N_e_s* of 5, a 10-fold reduction in *ρ* more than doubled the number of simulations in which both polymorphisms reached 50 *N_e_* generations (*P* < 10^−5^). At intermediate values of *N_e_s* the effect of *ρ* was quite strong, especially for higher values of *ρ*. At *N_e_s* of 10, fewer than 1% of simulations retained both polymorphisms for 50 *N_e_* generations if *ρ* was greater than or equal to 10, but 17% of simulations did so when *ρ* was 1, an increase of more than 20-fold (*P* < 10^−5^), and a majority of simulations retained both polymorphisms when *ρ* was less than or equal to 0.1. At higher values of *N_e_s*, the effect of *ρ* was again weaker, but still significant. At *N_e_s* of 20, 99% of simulations across all values of *ρ* less than 1 retained polymorphism at both loci for 50 *N_e_* generations, while only 84–94% simulations at values of *ρ* greater than 1 did so (*P* < 10^−3^).

### SLiM simulations

**Figure 4:**
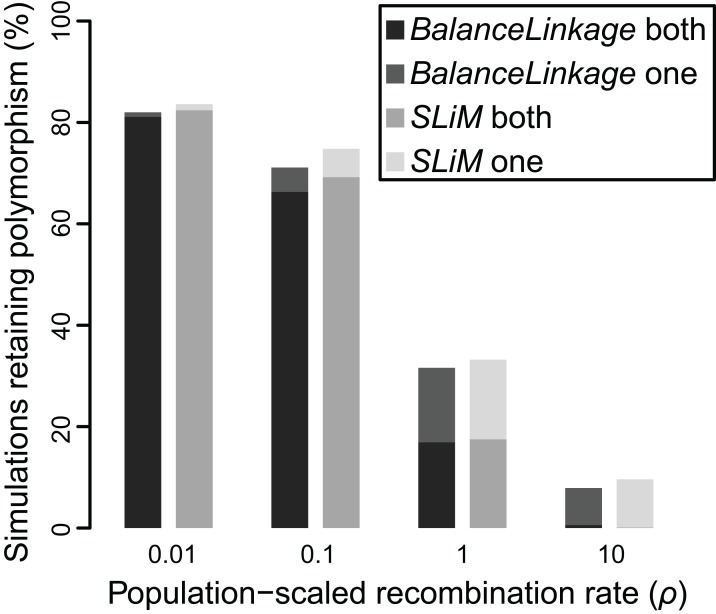
Proportion of simulations where polymorphism is retained after 50 *N*_e_ generations, under two independent methods, for *N*_e_*s* of 10 and recombination (ρ) ranging from 0.01 to 10. Greyscale shade indicates method (*BalanceLinkage* or *SLiM*) and results with either both or one polymorphism retained.

The results from *SLiM* were nearly identical to the results from *BalanceLinkage*. As before, weak selection (*N_e_s* = 5) resulted in retention of polymorphism for very few (≤2.5%) simulations regardless of recombination rate, while strong selection (*N_e_s* = 20) resulted in retention of polymorphism at both loci for most (>80%) simulations regardless of recombination rate. At intermediate values of selection (*N_e_s* = 10), there was a striking effect of recombination rate, nearly matching the results from *BalanceLinkage* (Fig. 4). For all parameter combinations examined, the difference between *SLiM* and *BalanceLinkage* in the number of simulations retaining polymorphism was less than 5% (Fig. 4). Thus, both methods capture the same phenomenon and confirm that linkage can have a substantial effect on whether two loci harbor adaptive polymorphism.

## Discussion

The fate of a non-neutral allele depends on the strength of selection and the force of genetic drift as determined by the effective population size. When these two evolutionary forces are close to evenly matched, linked polymorphism becomes an important cofactor. A locus under weak balancing selection may or may not retain its polymorphism, depending on whether the purifying effects of genetic drift win out over the effects of selection. However, two or more polymorphisms under weak balancing selection may enjoy a substantial selective advantage if linked to each other, because they can mutually boost each other’s adaptive effect. Thus, the combined strength of balancing selection maintaining two or more multi-locus haplotypes could be greater than the effect of either locus on its own. This may especially be true if selection is inconsistent, as polymorphism is likely to be lost during periods when selection does not act on it, unless it is linked to another polymorphism that is being maintained by selection. Similar to a “buddy system” in which two individuals give each other support and protection, pairs or groups of “buddy” polymorphisms can bolster each other. Alleles that would otherwise be swept away by drift are instead anchored by balancing selection. Thus, genetic diversity begets genetic diversity. Polymorphisms maintained for entirely unrelated reasons (e.g., immunity and reproductive strategy) may be more likely to be retained for long periods if they are closely linked.

Putatively balanced polymorphisms are often distributed non-randomly in the genome, but more research is required to empirically document the phenomenon described here. There are several cases where two or more closely-linked loci all show signatures of balancing selection, often with little evidence for epistasis. Some of these are complexes of structurally and functionally related genes, such as immune gene complexes (Takahata & Nei, 1990; Takahata, Satta & Klein, 1992; Makino & McLysaght, 2008; Tennessen et al., 2015; Tennessen, Bollmann & Blouin, 2017a) or plant R-gene complexes (Alcázar et al., 2014). In other cases, linked genes appear to have unrelated roles. Population genomic patterns have been most thoroughly characterized in humans, and the relatively low human effective population size (∼10^4^, Tenesa et al., 2007) suggests that low values of *N_e_s* and *ρ* may be common in our species. Thus, humans are an obvious system in which to test for linked balanced polymorphisms. For example, human genes *ARPC5* and *RGL1* are less than 1 kb apart, and both show some of the strongest signals of balancing selection in the genome, in populations across multiple continents (DeGiorgio, Lohmueller & Nielsen, 2014). Similarly, Bitarello et al. (2018) note that candidate balancing selection windows are non-randomly distributed across the human genome; while this pattern is driven by the major histocompatibility complex on Chromosome 6, their results also suggest other clusters, including one on Chromosome 10 overlapping *MYO3A* and *APBB1IP*. Unfortunately, it is difficult to discern if such cases are truly independent instances of balancing selection, or the population genetic signature of a single selected polymorphism plus surrounding neutral genetic variation (Siewert & Voight, 2017). Balancing selection signatures that are slightly farther apart are less likely to be artifacts of the same balanced polymorphism, but they are also less likely to be mutually supportive. For example, Andrés et al. (2009) identified 60 putative targets of balancing selection across the human genome, including several clusters of seemingly unrelated genes within 300 kb of each other (e.g., *LRAP* and *RIOK2*; *FUT2*, *PPP1R15A*, and *LHB*). In these cases, distances between linked genes exceed the typical width of LD blocks in humans (<100 kb, Reich et al., 2001), and do not show high LD in contemporary populations with LDlink (Machiela & Chanock, 2015), but are within the range of LD occasionally observed even in panmictic human populations (Koch, Ristroph & Kirkpatrick, 2013).

More work is needed to determine the extent to which genomic location influences the fate of an adaptive polymorphism under balancing selection. However, the results presented here suggest that the absence of epistasis is a plausible null hypothesis, and thus tight linkage is not sufficient evidence to conclude that two polymorphic genes interact with each other, even if they share a similar physiological role (e.g., immunological). For example, in *Biomphalaria* snails, the Guadeloupe Resistance Complex consists of several hyperdiverse genes with an apparent role in parasite recognition (Tennessen et al., 2015; Allan & Blouin, 2018; Allan et al., 2017a, 2017b), and these are closely linked to another variable locus, *sod1*, also influencing resistance to schistosome parasites (Goodall et al., 2004; Tennessen, Bollmann & Blouin, 2017a). While epistasis is an attractive explanation for such clustering of polymorphic, immune-relevant genes, they are not necessarily co-adapted. Similarly, sex chromosomes often undergo dynamic and seemingly adaptive restructuring. Translocation of the sex-determining region has occurred repeatedly in fishes (Lubieniecki et al., 2015), insects (Sharma et al., 2017), and flowering plants (Tennessen et al., 2017b). Such translocations may be favored in order to achieve tight linkage to other polymorphic loci; while such loci are often assumed to be under sexually antagonistic selection and thus in epistasis with the sex locus (Kirkpatrick, 2017), such interactions can’t be taken for granted. This may be especially true if dioecy offers little advantage over the hermaphroditic ancestral condition and thus balancing selection acting on the sex locus is relatively weak, as may be the case for wild strawberries (Spigler, Lewers & Ashman, 2011; Tennessen et al., 2017b).

## Conclusion

In summary, many balanced polymorphisms may owe their success to their genomic neighborhood, and not necessarily because of epistasis. Future research characterizing the phenotypic and fitness consequences of tightly linked polymorphisms will be invaluable for documenting and understanding this phenomenon in nature. Are linked adaptive polymorphisms retained by selection more often than solo polymorphisms, and thus disproportionate contributors to segregating adaptive variation? How often are chromosomal rearrangements, such as translocations or inversions, favored by selection because they facilitate tight linkage between functionally unrelated balanced polymorphisms? Is epistasis disproportionately prevalent within genomic regions showing high genetic diversity and/or LD? The results presented here suggest that linkage per se may be an important factor determining distributions and dynamics of adaptive genetic diversity.

## Supplemental Information

10.7717/peerj.5110/supp-1 Supplemental Information 1 Table S1. Median time to fixation (MTF) for combinations of *ρ* and *N_e_s*

## Additional Information and Declarations

### Data Availability

The following information was supplied regarding data availability: GitHub: https://github.com/jacobtennessen/GeneBuddies
