# Supplementary material for: Gene buddies: linked balanced polymorphisms reinforce each other even in the absence of epistasis
Source: PeerJ. 2018 Jun 28;6:e5110. doi: 10.7717/peerj.5110 (PMC6026533; doi:10.7717/peerj.5110)
Supplement: Supplemental Information 1 [file peerj-06-5110-s001.docx]

**Table S1. Median time to fixation (MTF) for combinations of *ρ* and *N_e_s*.**

| ***ρ*** | **0.005** | **0.01** | **0.02** | **0.05** | **0.1** | **0.2** | **0.5** | **1** | **2** | **5** | **10** | **20** |
| --- | --- | --- | --- | --- | --- | --- | --- | --- | --- | --- | --- | --- |
| ***N_e_s* = 4** | 6943 | 6607 | 6832 | 6773 | 6920 | 6461 | 5889 | 5636 | 5071 | 4656 | 4674 | 4150 |
| ***N_e_s* = 5** | 11024 | 12723 | 10936 | 10033 | 11070 | 10604 | 9590 | 7446 | 7539 | 5851 | 5407 | 5607 |
| ***N_e_s* = 6** | 21811 | 24759 | 21545 | 22607 | 20138 | 17692 | 14489 | 11778 | 9142 | 8153 | 6960 | 6697 |
| ***N_e_s* = 7** | 56251 | 48386 | 52633 | 44331 | 38751 | 28689 | 20341 | 17178 | 12922 | 10819 | 9855 | 8723 |
| ***N_e_s* = 8** | >10^5^ | >10^5^ | >10^5^ | 97880 | 69411 | 53923 | 31459 | 22845 | 19878 | 13788 | 12241 | 11594 |
| ***N_e_s* = 9** | >10^5^ | >10^5^ | >10^5^ | >10^5^ | >10^5^ | 95163 | 50660 | 34285 | 27621 | 18939 | 18066 | 17528 |
| ***N_e_s* = 10** | >10^5^ | >10^5^ | >10^5^ | >10^5^ | >10^5^ | >10^5^ | 74557 | 53379 | 38349 | 27272 | 24178 | 23306 |
| ***N_e_s* = 11** | >10^5^ | >10^5^ | >10^5^ | >10^5^ | >10^5^ | >10^5^ | >10^5^ | 74389 | 51649 | 37132 | 32340 | 30829 |
| ***N_e_s* = 12** | >10^5^ | >10^5^ | >10^5^ | >10^5^ | >10^5^ | >10^5^ | >10^5^ | >10^5^ | 75791 | 52260 | 44761 | 41390 |
| ***N_e_s* = 13** | >10^5^ | >10^5^ | >10^5^ | >10^5^ | >10^5^ | >10^5^ | >10^5^ | >10^5^ | >10^5^ | 77510 | 67397 | 60933 |
| ***N_e_s* = 14** | >10^5^ | >10^5^ | >10^5^ | >10^5^ | >10^5^ | >10^5^ | >10^5^ | >10^5^ | >10^5^ | >10^5^ | 90584 | 91288 |
| ***N_e_s* = 15** | >10^5^ | >10^5^ | >10^5^ | >10^5^ | >10^5^ | >10^5^ | >10^5^ | >10^5^ | >10^5^ | >10^5^ | >10^5^ | >10^5^ |
| ***N_e_s* = 16** | >10^5^ | >10^5^ | >10^5^ | >10^5^ | >10^5^ | >10^5^ | >10^5^ | >10^5^ | >10^5^ | >10^5^ | >10^5^ | >10^5^ |
| ***N_e_s* = 17** | >10^5^ | >10^5^ | >10^5^ | >10^5^ | >10^5^ | >10^5^ | >10^5^ | >10^5^ | >10^5^ | >10^5^ | >10^5^ | >10^5^ |
| ***N_e_s* = 18** | >10^5^ | >10^5^ | >10^5^ | >10^5^ | >10^5^ | >10^5^ | >10^5^ | >10^5^ | >10^5^ | >10^5^ | >10^5^ | >10^5^ |
| ***N_e_s* = 19** | >10^5^ | >10^5^ | >10^5^ | >10^5^ | >10^5^ | >10^5^ | >10^5^ | >10^5^ | >10^5^ | >10^5^ | >10^5^ | >10^5^ |
| ***N_e_s* = 20** | >10^5^ | >10^5^ | >10^5^ | >10^5^ | >10^5^ | >10^5^ | >10^5^ | >10^5^ | >10^5^ | >10^5^ | >10^5^ | >10^5^ |
